# Glutamate dehydrogenase activity and non-ınvasive fibrosis ındices (APRI, AAR, FIB-4) compared to FibroScan in chronic hepatitis B and hepatocellular carcinoma

**DOI:** 10.1007/s11845-026-04389-w

**Published:** 2026-04-17

**Authors:** Nuray Üremiş, Kadir Gişi, Teoman Şakalar, Fatma İnanç Tolun

**Affiliations:** 1 Department of Medical Biochemistry, Medical Faculty, Kahramanmaras Sutcu Imam University, Kahramanmaras, Turkey; 2 Department of Gastroenterology, Medical Faculty, Kahramanmaras Sutcu Imam University, Kahramanmaras, Turkey; 3 Department of Medical Oncology, Medical Faculty, Kahramanmaras Sutcu Imam University, Kahramanmaras, Turkey

**Keywords:** Fibrosis, Glutamate dehydrogenase, Hepatitis B, Hepatocellular carcinoma, Noninvasive indices

## Abstract

**Background:**

Glutamate dehydrogenase (GDH) levels are known to be important in predicting the development of complications associated with liver disease.

**Aim:**

In this study, we aimed to investigate the relationship between GDH activity and liver fibrosis grades, APRI, AAR, FIB-4 and AFP levels in patients with viral hepatitis B and hepatocellular carcinoma.

**Methods:**

The study group consisted of 95 individuals, including 65 HBV and 30 HCC patients, while the control group consisted of 45 healthy volunteers. Routine analyses, FibroScan evaluations, and non-invasive scoring were performed on the participants. GDH levels were determined using an ELISA kit.

**Results:**

Serum GDH activity was significantly higher in the HBV and HCC groups compared to the control group (*p* < 0.001). Similarly, AAR, FIB-4, and APRI values showed a progressive increase in parallel with disease progression. In patients with chronic HBV infection, GDH, AAR, FIB-4, and APRI levels increased markedly with advancing fibrosis stages. In HCC cases, these parameters were also significantly elevated in the advanced fibrosis stage (F4) (*p* < 0.01 and *p* < 0.001). GDH demonstrated a high diagnostic performance, with an AUC of 0.808 in HCC. Correlation analysis revealed a significant positive association between GDH and AST levels in the HBV group (r = 0.35, *p* = 0.01). Moreover, FIB-4 and APRI indices showed strong positive correlations with AST, GGT, and LDH levels.

**Conclusions:**

These findings suggest that GDH, in conjunction with non-invasive fibrosis indices, may serve as a potential biomarker reflecting hepatic injury and fibrosis severity.

## Introduction

Liver fibrosis is a process that causes structural damage and loss of function in the liver as a result of excessive accumulation of collagen and other matrix proteins in response to inflammation [1, 2]. Therefore, determining the degree of fibrosis is essential in predicting the prognosis of chronic liver diseases, planning treatment strategies, and selecting appropriate patients for transplantation [3, 4]. The degree of fibrosis is used as a clinical indicator reflecting the severity and progression rate of the disease [5]. In individuals with chronic Hepatitis B virus (HBV) infection, the degree of fibrosis is an essential criterion in treatment management and evaluation of response to antiviral therapy [6], and it also plays a critical role in predicting the development of hepatocellular carcinoma (HCC) [7]. In addition, the AST Platelet Ratio Index (APRI), AST/ALT Ratio (AAR), and Fibrosis-4 (FIB-4) index are widely used biochemical indices for the non-invasive assessment of chronic liver diseases [8]. They have been incorporated into clinical practice, particularly in the evaluation of progressive liver damage such as liver fibrosis [9]. Both indices are essential in predicting the degree of liver fibrosis using non-invasive methods and serve as alternatives or supplements to liver biopsy in clinical practice [10].

The liver is an organ that plays a central role in protein breakdown and detoxification processes [11]; glutamate dehydrogenase (GDH) is a critical enzyme in these metabolic events [12]. GDH contributes to the TCA cycle by participating in amino acid metabolism and mediates energy production and ammonia detoxification [13]. Additionally, GDH is clinically significant due to its functions related to alanine aminotransferase (ALT), aspartate aminotransferase (AST), and alpha-fetoprotein (AFP), which are commonly used in liver function tests [14, 15]. Although ALT, AST, and AFP are used as gold standard biomarkers in the diagnosis of various liver diseases, their limited liver specificity necessitates the identification of new and more sensitive biomarkers s[16–20]. In this context, it is anticipated that serum GDH levels may reflect liver tissue-related diseases and metabolic imbalances [21]. This study aims to evaluate the relationship between GDH activity and liver fibrosis grades, APRI, AAR, and AFP levels in patients diagnosed with hepatitis B infection and HCC. In this regard, the role of GDH as a potential biomarker for the more sensitive and comprehensive evaluation of hepatic pathologies has been investigated.

## Patients and methods

### Study population

This study included patients who visited the Gastroenterology and Oncology outpatient clinics at KSU Hospital. The study group consisted of a total of 95 individuals, including 65 patients diagnosed with HBV infection and 30 patients diagnosed with HBV-related hepatocellular carcinoma. The control group consisted of 45 healthy volunteers without systemic disease. The study was approved by the KSU Medical Research Ethics Committee under protocol number 2024/33–314. Inclusion criteria included being between 30 and 80 years of age, having a diagnosis of HBV infection, and having HBV-related HCC. All HBV and HCC cases were selected from individuals who had not yet received treatment and had recently been diagnosed. Written informed consent was obtained from all individual participants included in the study.

### Fibroscan analysis

A transient elastography device (Echosens FibroScan® 502) was used to assess liver fibrosis and parenchymal stiffness levels in all participants. Elastography was used to quantitatively measure liver tissue stiffness and fat content (CAP). Measurements were performed using an ultrasound probe placed in the right lobe of the patients through the intercostal space. The median value (E-median) and CAP values indicating the degree of fibrosis were recorded for each individual. The data obtained were used in statistical analyses to evaluate the relationship between liver function tests and liver fibrosis.

### Biochemical analyses

In routine biochemical analyses of patients, haematological parameters such as HGB, PLT, and WBC were evaluated from whole blood samples. In contrast, ALT, AST, GGT, and AFP levels were assessed from serum samples. Blood samples taken in gel biochemistry tubes were centrifuged at 4000 rpm for 5 min. The serum was separated, and the samples were aliquoted and stored for GDH analysis. Non-invasive scores based on routine biochemical parameters were calculated to assess liver fibrosis. The AST/ALT ratio (AAR) was determined as the ratio of serum AST (U/L) to ALT (U/L). The AST-to-platelet ratio index (APRI) and Fibrosis-4 (FIB-4) index were calculated using the following formulas.

$$APRI= \left(\frac{AST\left(U/L\right)}{Upper limit of normal \left(ULN\right) forAST} \right) \times \left(\frac{100}{Platelet count \left({\times 10}^{9}/L\right)}\right)$$

$$FIB-4=\frac{Age(years)*AST (U/L)}{\text{Platelet count }\left(\times \frac{{10}^{9}}{\mathrm{L}}\right)\mathrm{X}\sqrt{ALT(U/L)}}$$

### Measurement of serum GDH/GLDH levels

Glutamate dehydrogenase (GDH/GLDH) levels in patients’ serum samples were quantitatively measured using a commercial ELISA kit (Elabscience EL-H6068) by the manufacturer’s protocol. The measurement process was carried out as follows: Samples were added to a 96-well microtitre plate along with the standards and blank controls provided by the kit and incubated. Then, biotin-labelled detection antibody was added, and the plates were incubated. Subsequently, a working solution containing HRP-conjugate was added, and incubation was performed at 37°C for 30 min. After incubation with the substrate solution for 15 min in the dark, the reaction was stopped by adding the stop solution. Absorbance was read at 450 nm. The absorbance values obtained were calculated according to the standard curve to determine the GDH level (mIU/mL) of each sample.

### Statistical analysis

Statistical analyses were performed using GraphPad Prism version 10.6.1. Data normality was assessed with the Shapiro–Wilk test, and variance homogeneity was evaluated before applying the appropriate tests. Since biochemical and hematological data among the Control, HBV, and HCC groups were not normally distributed, the Kruskal–Wallis test followed by Dunn’s multiple comparison test was used. For fibrosis-based subgroup analyses, HBV patients were classified as F0–F1, F2–F3, and F4, and comparisons among these subgroups were conducted using one-way ANOVA with Tukey’s post-hoc test. In the HCC group, due to the absence of F0–F1 cases, comparisons between the remaining two subgroups were performed using an independent-samples Student’s t-test. Correlations between biochemical and clinical parameters in HBV and HCC groups were evaluated using the Spearman rank correlation test. Receiver operating characteristic (ROC) analyses were performed for GDH, APRI, AAR, FIB-4, AFP, ALT, and AST to assess their diagnostic performance between Control vs. HBV and Control vs. HCC groups. AUC values with 95% confidence intervals, sensitivity, specificity, and optimal cut-off points were obtained based on Youden’s index. A *p* value < 0.05 indicated statistical significance. Data are reported as mean ± SD.

## Results

### Clinical characteristics

Routine biochemical analyses revealed significant differences in liver function tests among the study groups (Table 1). Serum AST, GGT, ALP, and LDH levels were significantly higher in the HCC group compared with both the healthy control and HBV groups (all *p* < 0.001). In contrast, ALT levels were lower in the HCC group than in the HBV group (control–HCC *p* < 0.001; control–HBV *p* < 0.02), suggesting a possible decline in hepatocellular enzyme activity as the disease progresses. Serum albumin concentrations were significantly lower in both the HBV and HCC groups compared with the control group (*p* < 0.001 for both), with the lowest levels observed in the HCC group (HBV–HCC *p* = 0.02). This finding indicates a reduction in the liver’s protein synthesis capacity with increasing disease severity. Ferritin and LDH levels showed a gradual increase from the control to the HBV and HCC groups (*p* < 0.001 for all), which may reflect enhanced cellular damage as the disease advances. Similar deterioration was observed in coagulation parameters: prothrombin time percentage (PT%) values were significantly decreased, whereas INR values were increased in both the HBV and HCC groups compared with the control group. Furthermore, disease-related changes were also evident in hematological indices. Hemoglobin, HCT, and PLT counts were significantly lower in the HBV and HCC groups compared with the control group (*p* < 0.05), whereas RDW was significantly higher. Importantly, AFP levels were markedly elevated in the HCC group compared with both the control and HBV groups (*p* < 0.001), supporting the high diagnostic and prognostic value of AFP in HCC.

**Table 1:** Biochemical parameters of healthy control group, hepatitis B (HBV) and hepatocellular carcinoma (HCC) patients

	Control Mean ± SD	HBV Mean ± SD	HCC Mean ± SD	*p-v*alue Control vs. HBV	*p-v*alue Control vs. HCC	*p-v*alue HBV vs. HCC
*n* Sex (M/F)	50 28/22	65 30/35	30 18/12			
ALT (U/L)	25.8 ± 7.47	21.7 ± 7.38	17.5 ± 4.48	**0.02**	** < 0.001**	0.14
AST (U/L)	18 ± 5.66	19.1 ± 6.47	52.1 ± 26.1	> 0.99	** < 0.001**	** < 0.001**
GGT (U/L)	42.9 ± 19.2	35.6 ± 25.4	141 ± 47.1	**0.03**	** < 0.001**	** < 0.001**
ALP (U/L)	75.2 ± 19.1	84.2 ± 20.7	194 ± 35.9	0.14	** < 0.001**	** < 0.001**
LDH (U/L)	178 ± 30.7	207 ± 45.0	316 ± 73.5	**0.009**	** < 0.001**	** < 0.001**
Ferritin (µg/L)	94.1 ± 35.2	119 ± 39.1	245 ± 84.9	**0.03**	** < 0.001**	** < 0.001**
Albumin (g/L)	46.2 ± 2.13	42.2 ± 4.80	36.1 ± 7.27	** < 0.001**	** < 0.001**	**0.02**
RBC (10^6/µL)	5.12 ± 0.45	5.39 ± 5.06	4.48 ± 1.19	**0.04**	0.06	> 0.99
Hemoglobin (g/dL)	14.9 ± 1.37	13.8 ± 2.31	12.7 ± 2.93	0.07	**0.03**	0.75
HCT (%)	43.9 ± 3.37	40.8 ± 5.69	38.6 ± 8.77	**0.01**	**0.004**	0.33
MCV (fL)	85.9 ± 4.53	86.2 ± 6.68	86.8 ± 7.31	> 0.99	> 0.99	> 0.99
MCH (pg)	29.1 ± 1.96	29.0 ± 3.11	28.8 ± 3.78	> 0.99	> 0.99	> 0.99
RDW (fl)	40.6 ± 2.60	44.0 ± 5.92	51.7 ± 9.39	**0.01**	** < 0.001**	**0.01**
PLT (10^9/L)	265 ± 41.9	232 ± 54.7	227 ± 62.2	**0.003**	**0.03**	0.93
PT (%)	99.7 ± 13.3	88.2 ± 21.5	66.2 ± 18.5	**0.006**	** < 0.001**	**0.006**
INR	1.02 ± 0.09	1.11 ± 0.17	1.39 ± 0.33	**0.04**	** < 0.001**	**0.003**
PT (Sec)	12.0 ± 1.23	12.7 ± 1.82	25.9 ± 39.9	0.27	** < 0.001**	**0.008**
APTT (Sec)	25.2 ± 3.64	25.9 ± 3.78	30.1 ± 7.31	> 0.99	**0.09**	0.12
AFP (IU/mL)	2.75 ± 1.57	3.92 ± 2.22	960 ± 214	**0.01**	** < 0.001**	** < 0.001**

*ALT* Alanine aminotransferase, *AST* Aspartate aminotransferase, *GGT* Gamma-glutamyl transferase, *ALP* Alkaline phosphatase, *LDH* Lactate dehydrogenase, *RBC* Red blood cell count, *HCT* Hematocrit, *MCV* Mean corpuscular volume, *MCH* Mean corpuscular hemoglobin, *RDW* Red cell distribution width, *PLT* Platelet count, *PT* Prothrombin time, *INR* International normalized ratio, *APTT* Activated partial thromboplastin time, *AFP* Alpha-fetoprotein.

Data are presented as Mean ± SD. Comparisons among Control, HBV, and HCC groups were performed using one-way ANOVA followed by Tukey’s post hoc test for normally distributed variables, and the Kruskal–Wallis test followed by Dunn’s post hoc test for non-normally distributed variables. Statistically significant results (*p* < 0.05) are indicated in bold.

### Evaluation of GDH, APRI, AAR, and FIB-4 levels in liver disease progression

**Fig. 1:**
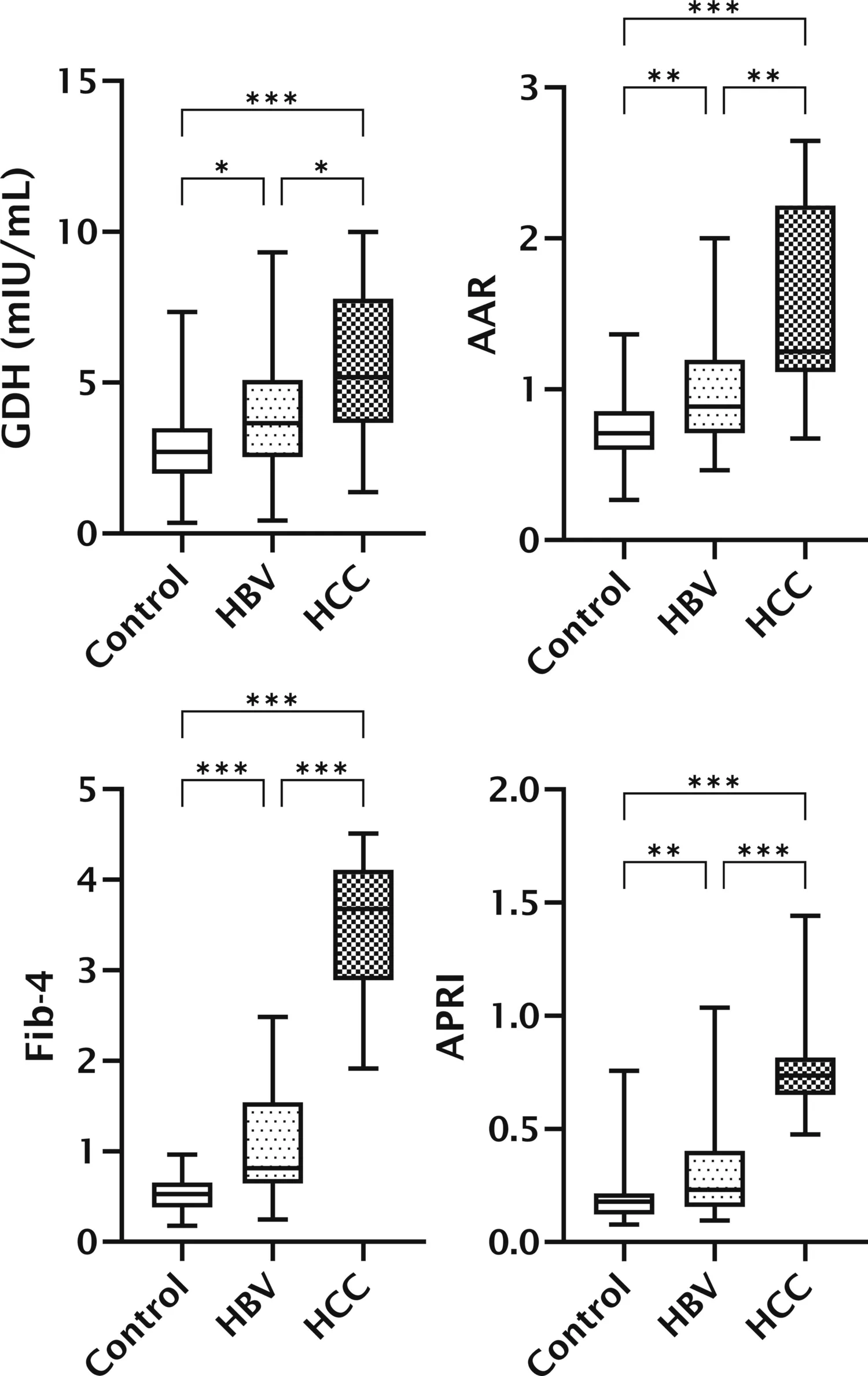
Comparison of GDH, AAR, FIB-4, and APRI levels in HBV and HCC patients with control group. HBV (*n* = 65), HCC (*n* = 30), and control group (*n* = 45).Data are presented as mean ± SD. *p < 0.05 was considered statistically significant. Abbreviations: GDH, glutamate dehydrogenase; AAR, AST-to-ALT ratio; FIB-4, fibrosis-4 index; APRI, AST-to-platelet ratio index

In this study, serum GDH levels and non-invasive liver fibrosis indices were compared among healthy controls, patients with chronic HBV infection, and those with HCC (Fig. 1). GDH levels were significantly elevated in the HBV group compared with the control group (*p* < 0.05). This increase was more pronounced in the HCC group, showing a highly significant difference compared with the control group (*p* < 0.001). Furthermore, GDH levels in the HCC group were significantly higher than those in the HBV group (*p* < 0.05). These findings suggest that GDH may be closely associated with the progression of hepatocellular injury. AAR levels were lowest in the control group and highest in the HCC group, with statistically significant differences between all groups (control vs. HBV: *p* < 0.01; HBV vs. HCC: *p* < 0.01; control vs. HCC: *p* < 0.001). Similarly, the FIB-4 index increased in direct proportion to disease severity, with significant differences observed among the control, HBV, and HCC groups (*p* < 0.001). This finding indicates that the FIB-4 index strongly correlates with the degree of liver fibrosis and the progression toward HCC. APRI levels also showed a gradual increase parallel to disease advancement. Significant elevations were detected in both the HBV (*p* < 0.01) and HCC (*p* < 0.001) groups compared with the control group. Moreover, APRI levels in the HCC group were significantly higher than those in the HBV group (*p* < 0.001). Overall, GDH, AAR, FIB-4, and APRI levels showed a significant increase parallel to the progression of liver disease. These findings support the potential use of these parameters as biomarkers, particularly in monitoring HBV infection and HCC development.

### The relationship between fibrosis stage and GDH, APRI, AAR, and FIB-4 levels in chronic hbv infection

**Fig. 2:**
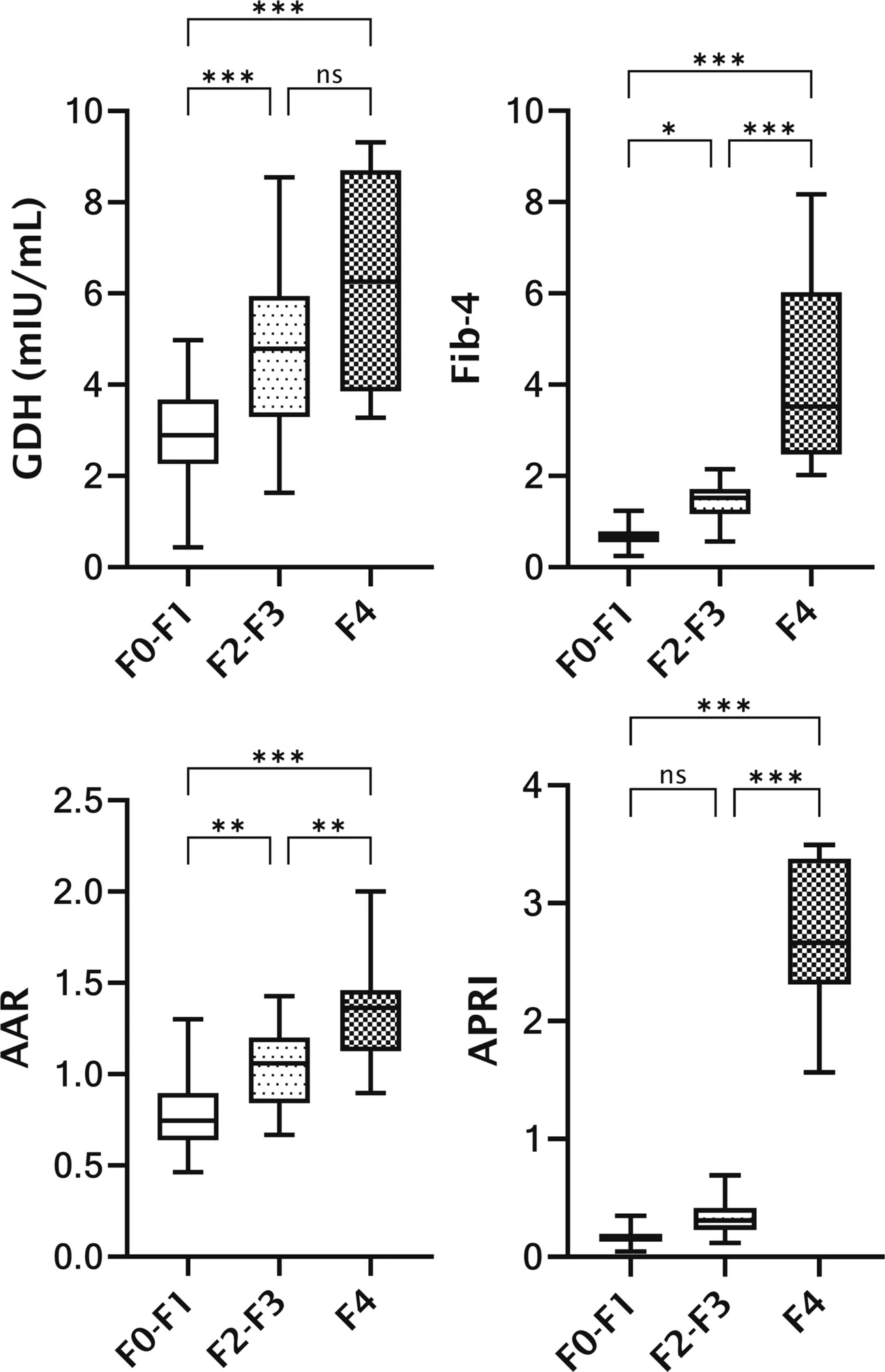
Relationship between fibrosis stage and GDH, FIB-4, AAR, and APRI Levels in Chronic HBV Infection (*n* = 65). Data are presented as mean ± SD. Differences between fibrosis stages were analyzed, and *p < 0.05 was considered statistically significant. Abbreviations: GDH, glutamate dehydrogenase; AAR, AST-to-ALT ratio; FIB-4, fibrosis-4 index; APRI, AST-to-platelet ratio index

Significant increases in serum GDH, APRI, AAR, and FIB-4 values were observed with advancing fibrosis stage in patients with chronic HBV infection (Fig. 2). GDH levels were significantly higher in the F2–F3 and F4 groups compared with the F0–F1 group (*p* < 0.001). A clear upward trend in FIB-4 scores was also detected across fibrosis stages, with significant differences among the F0–F1, F2–F3, and F4 groups (*p* < 0.05, *p* < 0.001). Similarly, AAR values increased as fibrosis progressed, showing significant elevations in both the F2–F3 and F4 groups compared with the F0–F1 group (*p* < 0.01). Regarding APRI scores, a significant increase was observed in the F4 group compared with the F0–F1 group (*p* < 0.001). Although no significant difference was found between the F0–F1 and F2–F3 groups (*p* > 0.05), the difference between the F2–F3 and F4 groups was statistically significant (*p* < 0.001). These findings indicate that serum GDH levels and non-invasive fibrosis indices increase in parallel with fibrosis progression and may therefore reflect the degree of liver fibrosis in chronic HBV infection.

### The relationship between fibrosis stage and GDH, APRI, AAR, and FIB-4 levels in HCC

**Fig. 3:**
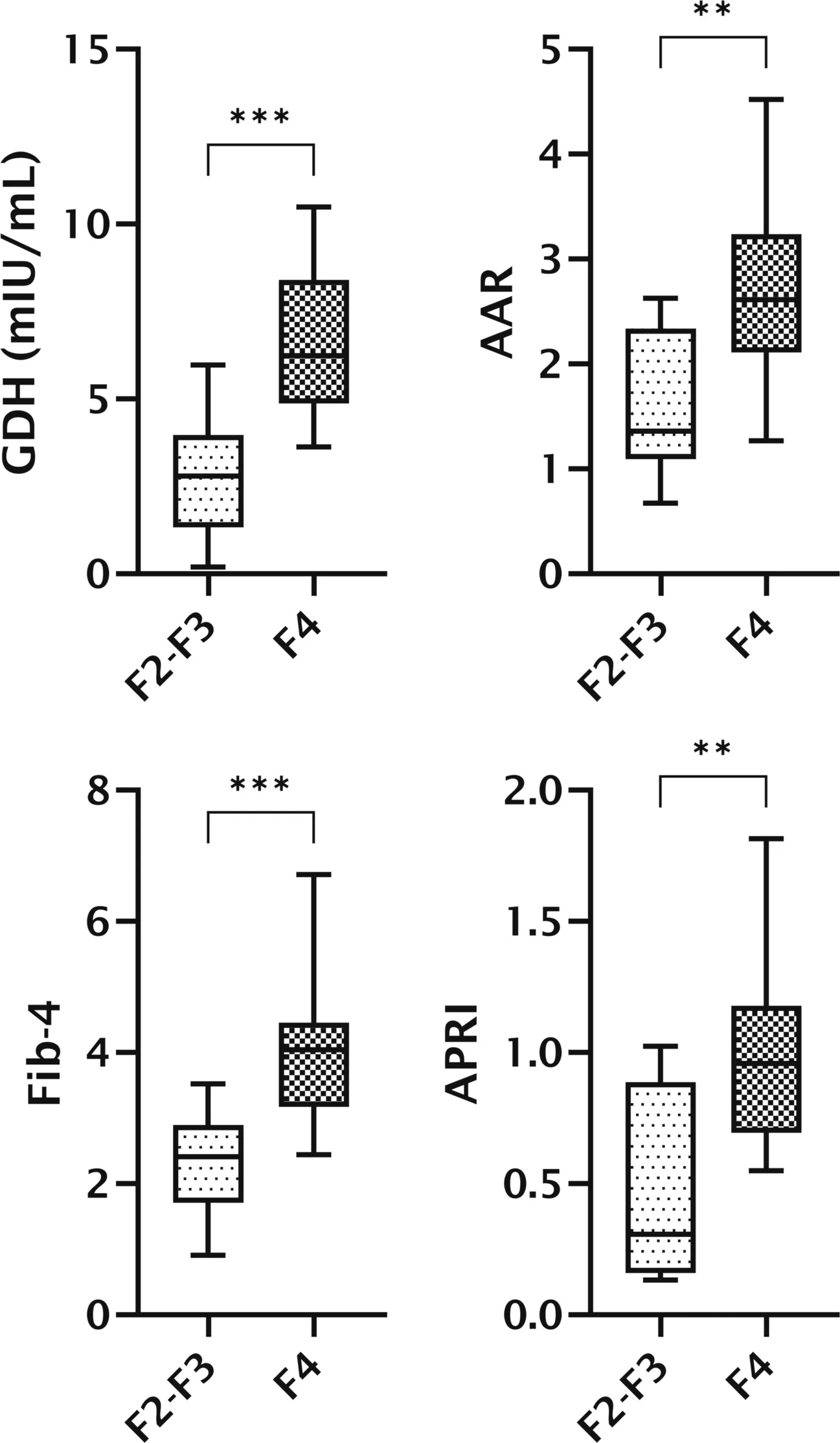
Relationship between fibrosis stage and GDH, FIB-4, AAR, and APRI levels in HCC (*n* = 30). Data are presented as mean ± SD. Differences between fibrosis stages were analyzed, and *p < 0.05 was considered statistically significant. Abbreviations: GDH, glutamate dehydrogenase; AAR, AST-to-ALT ratio; FIB-4, fibrosis-4 index; APRI, AST-to-platelet ratio index

In patients with HCC, significant increases in serum GDH and non-invasive fibrosis indices were observed in parallel with advancing fibrosis stage (Fig. 3). GDH levels were significantly higher in the F4 group compared with the F2–F3 group (*p* < 0.001). FIB-4 scores showed a similar significant increase in the F4 group (*p* < 0.001). AAR values also tended to rise with fibrosis progression and were significantly higher in the F4 group compared with the F2–F3 group (*p* < 0.01). Furthermore, a significant increase in APRI scores was observed in the F4 group (*p* < 0.01). These findings indicate that the advanced fibrosis stage (F4) in patients with HBV-related HCC is closely associated with elevated serum GDH levels and non-invasive fibrosis indices.

### Correlation between GDH and liver function tests

The association between serum GDH concentrations and standard liver function tests in HBV-infected patients was examined using Spearman’s correlation (Table 2). The analysis revealed a significant positive correlation between GDH levels and AST (r = 0.352, *p* = 0.009). In contrast, no significant correlations were observed between GDH levels and ALT, GGT, ALP, LDH, or AFP. In the HCC group, no significant correlations were found between GDH levels and any of the biochemical parameters examined (*p* > 0.05) (Table 3).

**Table 2:** Spearman correlations between glutamate dehydrogenase (GDH) and selected biochemical parameters in HBV patients

	GDH vs. ALT	GDH vs. AST	GDH vs. GGT	GDH vs. ALP	GDH vs. LDH	GDH vs. AFP
Spearman r	0.078	0.352	0.014	−0.039	−0.002	−0.137
*P* value	0.573	0.009	0.916	0.776	0.992	0.293
Significant (alpha = 0.05)	No	Yes	No	No	No	No

Spearman’s rank correlation coefficients (r) and two-tailed p-values are shown for correlations between GDH and ALT, AST, GGT, ALP, LDH, and AFP in patients with hepatitis B virus (HBV) infection. “Significant” indicates *p* < 0.05 (α = 0.05). Analyses were performed using Spearman’s rank correlation.

Abbreviation: *GDH* Glutamate dehydrogenase, *ALT* Alanine aminotransferase, *AST* Aspartate aminotransferase, *GGT* Gamma-glutamyl transferase, *ALP* Alkaline phosphatase, *LDH* Lactate dehydrogenase, *AFP* Alpha-fetoprotein.

**Table 3:** Spearman correlations between glutamate dehydrogenase (GDH) and selected biochemical parameters in HCC patients

	GDH vs. ALT	GDH vs. AST	GDH vs. GGT	GDH vs. ALP	GDH vs. LDH	GDH vs. AFP
Spearman r	0.050	0.346	0.050	0.264	0.086	−0.200
*P* value	0.879	0.206	0.854	0.359	0.763	0.456
Significant (alpha = 0.05)	No	No	No	No	No	No

Spearman’s rank correlation coefficients (r) and two-tailed p-values are shown for correlations between GDH and ALT, AST, GGT, ALP, LDH, and AFP in patients with hepatitis B virus (HCC) infection. “Significant” indicates *p* < 0.05 (α = 0.05). Analyses were performed using Spearman’s rank correlation.

Abbreviation: *GDH* Glutamate dehydrogenase, *ALT* Alanine aminotransferase, *AST* Aspartate aminotransferase, *GGT* Gamma-glutamyl transferase, *ALP* Alkaline phosphatase, *LDH* Lactate dehydrogenase, *AFP* Alpha-fetoprotein.

### Correlation between non-invasive fibrosis indices and liver function tests

The correlations between liver function tests and non-invasive fibrosis indices were evaluated in patients diagnosed with HBV and HCC (Table 4). In the HBV group, a significant negative correlation was observed between ALT and AAR (r = − 0.332, *p* = 0.013), whereas a significant positive correlation was found between ALT and APRI (r = 0.293, *p* = 0.033). Furthermore, significant positive correlations were identified between AST and FIB-4 (r = 0.448, *p* = 0.001) and between AST and APRI (r = 0.582, *p* < 0.001). GGT levels also showed significant positive correlations with FIB-4 (r = 0.389, *p* = 0.004) and APRI (r = 0.434, *p* < 0.001). A statistically significant correlation was additionally observed between LDH and APRI (r = 0.304, *p* = 0.035). In the HCC group, no statistically significant correlations were found between the fibrosis indices and liver function tests or AFP levels. These findings indicate that the FIB-4 and APRI indices are closely associated with changes in liver enzyme levels, particularly in patients with HBV infection.

**Table 4:** Evaluation of the correlation of AAR, Fib-4 and APRI with liver function tests and AFP levels in HBV, and HCC patients

		HBV			HCC		
		AAR	Fib-4	APRI	AAR	Fib-4	APRI
ALT	*r*	−0.332	0.130	0.293	−0.368	0.171	0.132
	p	**0.013**	0.369	**0.033**	0.327	0.592	0.682
AST	*r*	0.139	0.448	0.582	−0.077	−0.082	−0.339
	*p*	0.320	**0.001**	** < 0.001**	0.817	0.773	0.216
GGT	*r*	−0.111	0.389	0.434	0.358	0.370	−0.120
	*p*	0.400	**0.004**	** < 0.001**	0.229	0.174	0.669
ALP	*r*	0.002	0.235	0.152	0.242	−0.225	−0.264
	*p*	0.988	0.113	0.283	0.446	0.459	0.383
LDH	*r*	0.126	0.267	0.304	−0.021	0.071	0.054
	*p*	0.395	0.084	**0.035**	0.956	0.803	0.852
AFP	*r*	−0.033	−0.009	0.029	−0.077	−0.004	−0.089
	*p*	0.802	0.950	0.825	0.806	0.995	0.753

Spearman’s rank correlation coefficients (r) and two-tailed p-values are shown for correlations of AAR, FIB-4, and APRI with ALT, AST, GGT, ALP, LDH, and AFP in patients with HBV and HCC. Statistically significant results (*p* < 0.05) are indicated in bold.

Abbreviation: *AAR* AST to ALT ratio, *FIB-4* Fibrosis-4 index, *APRI* AST Platelet Ratio Index, *ALT* Alanine aminotransferase, *AST* Aspartate aminotransferase, *GGT* Gamma-glutamyl transferase, *ALP* Alkaline phosphatase, *LDH* Lactate dehydrogenase, *AFP* Alpha-fetoprotein, *HBV* Hepatitis B virus, *HCC* Hepatocellular carcinoma.

### Diagnostic values of ALT, AST, GDH, AFP, APRI, AAR, and FIB-4 in chronic HBV infection and HCC

**Fig. 4:**
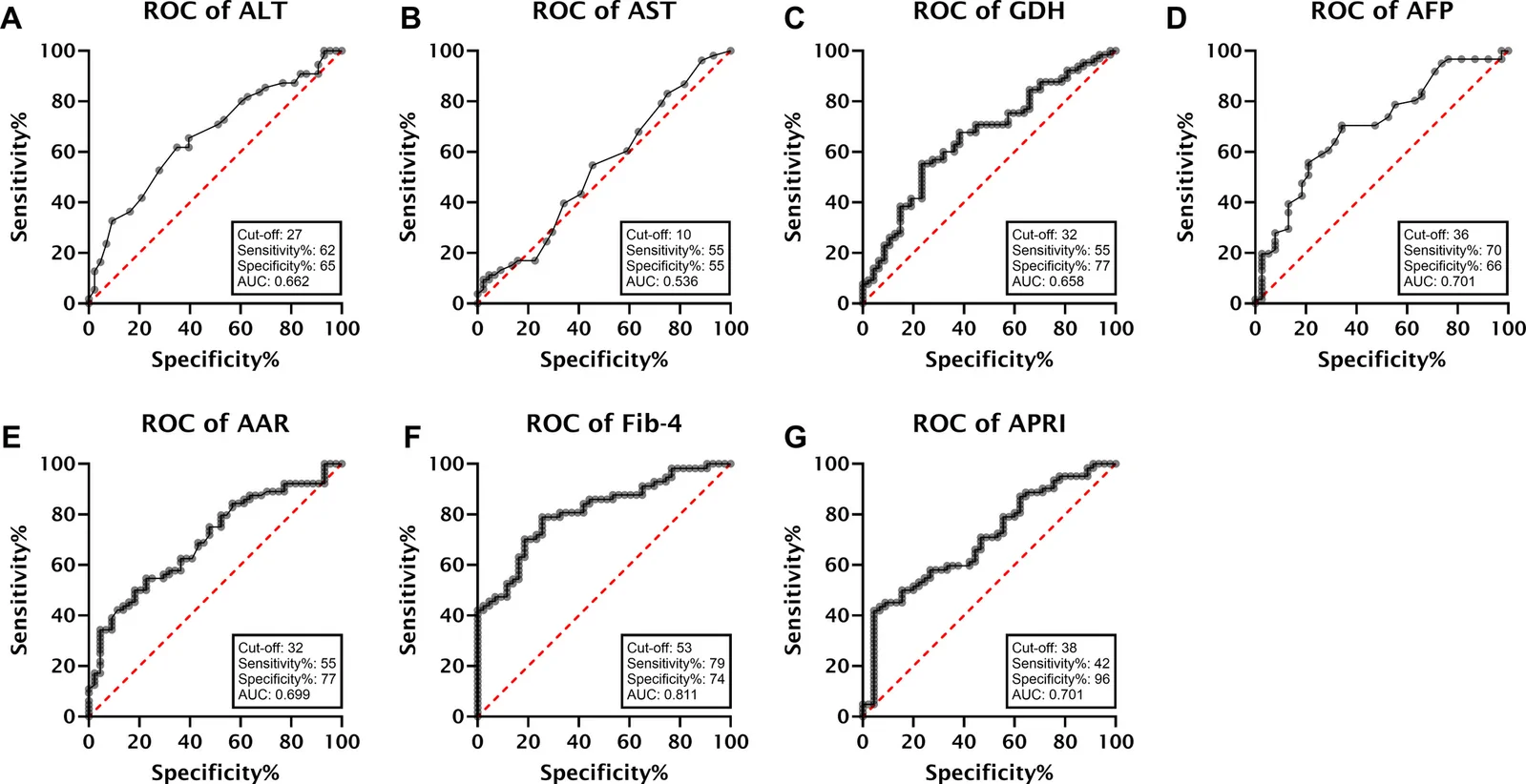
ROC analyses of ALT (**A**), AST (**B**), GDH (**C**), AFP (**D**), AAR (**E**), FIB-4 (**F**), and APRI (**H**) in HBV patients. HBV (*n* = 65) and control group (*n* = 45). Area under the curve (AUC), sensitivity, and specificity values were calculated. *p < 0.05 was considered statistically significant. Abbreviations: ALT, alanine aminotransferase; AST, aspartate aminotransferase; GDH, glutamate dehydrogenase; AFP, alpha-fetoprotein; AAR, AST-to-ALT ratio; FIB-4, fibrosis-4 index; APRI, AST-to-platelet ratio index

The ROC analyses demonstrated that the evaluated biomarkers exhibited moderate diagnostic performance in the chronic HBV group. ALT showed a modest discriminative ability with an AUC of 0.662, whereas AST displayed limited performance (AUC 0.536) (Fig. 4). The AUC value of GDH (0.658) indicated a meaningful contribution to detecting hepatocellular injury associated with HBV infection. AFP exhibited an intermediate level of diagnostic accuracy with an AUC of 0.701. Fibrosis indices demonstrated stronger diagnostic performance in the HBV group. AAR (AUC 0.699) and APRI (AUC 0.701) provided moderate discriminative capacity, while FIB-4 (AUC 0.811) achieved the highest accuracy and emerged as the most reliable non-invasive parameter for fibrosis assessment in HBV patients.

**Fig. 5:**
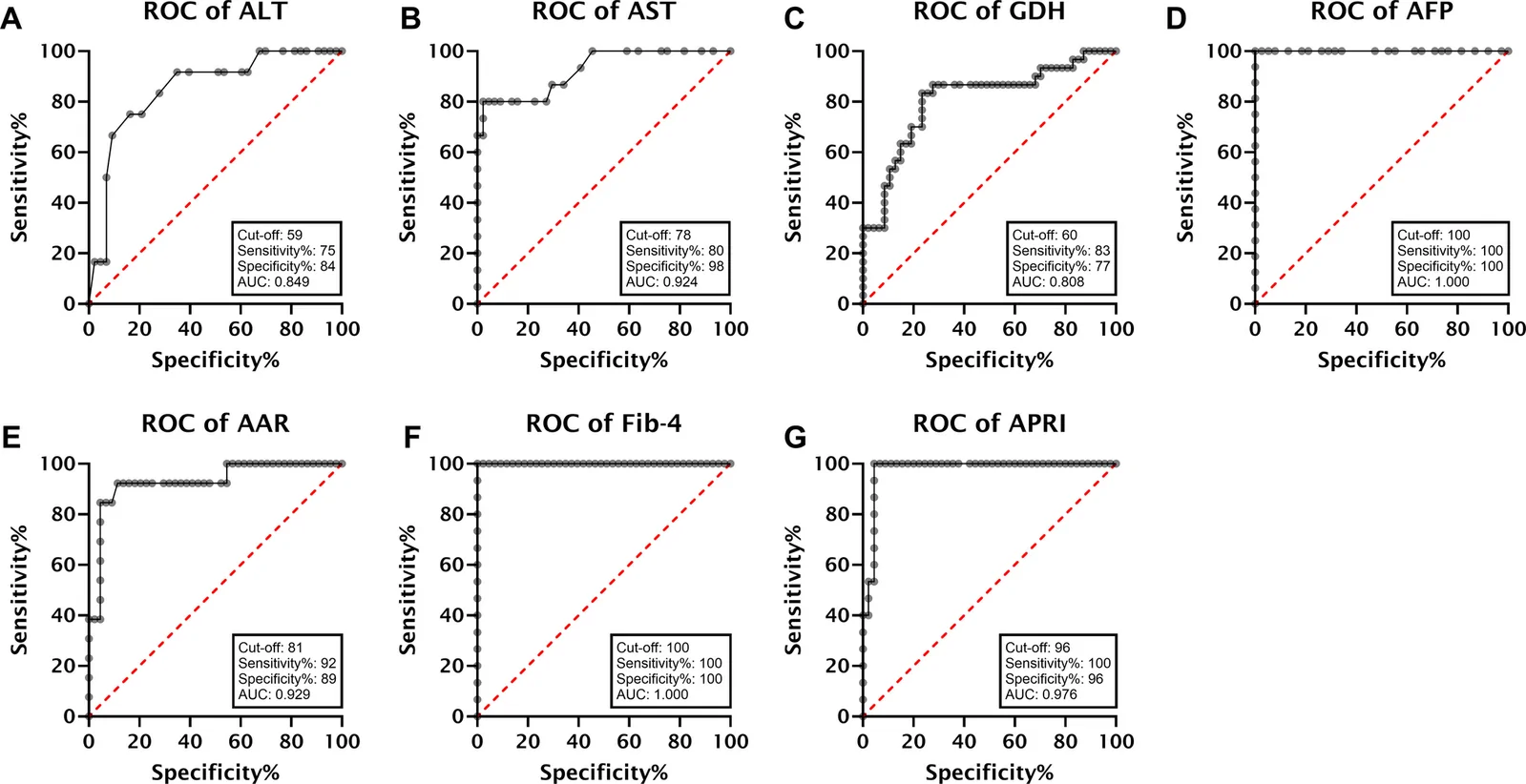
ROC analyses of ALT (**A**), AST (**B**), GDH (**C**), AFP (**D**), AAR (**E**), FIB-4 (**F**), and APRI (**H**) in HCC patients. HCC (*n* = 30) and control group (*n* = 45). Area under the curve (AUC), sensitivity, and specificity values were calculated. *p < 0.05 was considered statistically significant. Abbreviations: ALT, alanine aminotransferase; AST, aspartate aminotransferase; GDH, glutamate dehydrogenase; AFP, alpha-fetoprotein; AAR, AST-to-ALT ratio; FIB-4, fibrosis-4 index; APRI, AST-to-platelet ratio index

In the HCC group, the diagnostic strength of the biomarkers increased markedly. ALT (AUC 0.849) and particularly AST (AUC 0.924) exhibited high discriminative power (Fig. 5). GDH also demonstrated robust diagnostic performance with an AUC of 0.808. Fibrosis indices showed exceptionally high accuracy in HCC. AAR (AUC 0.929) performed strongly, whereas FIB-4 (AUC 1.000) and APRI (AUC 0.976) approached the diagnostic capacity of AFP, the gold-standard biomarker for hepatocellular carcinoma (AUC 1.000). Overall, FIB-4 demonstrated high diagnostic performance in both HBV and HCC groups, while APRI showed superior accuracy particularly in HCC. GDH activity provided meaningful diagnostic value across both clinical conditions.

## Discussion

This study aims to evaluate the pathophysiological events that cause liver damage in the course of HBV infection and hepatocellular carcinoma in light of GDH levels, APRI/AAR indices, AFP levels, and fibrosis development mechanisms. In particular, the role of serum GDH levels in determining the prognosis of hepatic diseases and predicting the development of complications associated with chronic liver diseases has been discussed.

In this study, conventional routine biochemical and hematological parameters, including HGB, PLT, WBC, ALT, AST, GGT, and AFP, were comprehensively evaluated alongside GDH and fibrosis indices. Hemoglobin levels were found to be lower in patients with HCC compared to the control group, which may reflect anemia associated with chronic disease and impaired liver function. Platelet counts were significantly decreased in both HBV and HCC groups, supporting the well-established association of this parameter with liver fibrosis and portal hypertension. Regarding liver enzymes, ALT levels were slightly decreased in HBV and HCC patients, whereas AST and GGT levels were markedly elevated—particularly in the HCC group. This pattern is consistent with advanced liver injury and impaired hepatocellular integrity. The disproportionate increase in AST relative to ALT may indicate mitochondrial damage and progression of fibrosis, which is also in line with the elevated GDH levels observed. Similarly, the significant increase in GGT levels in HCC patients further supports the presence of cholestasis and tumor-associated hepatic dysfunction. The markedly elevated AFP levels observed in the HCC group are consistent with tumor burden and disease progression. Overall, these findings, when evaluated in conjunction with GDH and non-invasive fibrosis indices, provide a more comprehensive understanding of disease severity in patients with HBV and HCC.

GDH plays a central role in amino acid catabolism through oxidative deamination reactions [21]. This enzyme converts glutamate into α-ketoglutarate and ammonia (NH₃) in the presence of NADP(H) and/or NAD(H), enabling the carbon skeleton to be used for energy production [22]. The resulting α-ketoglutarate is integrated into energy metabolism by entering the tricarboxylic acid (TCA) cycle, while the remaining ammonia is detoxified by entering the urea cycle in the liver [23]. Its role as a central enzyme in amino acid metabolism, formed by the breakdown of proteins, its contribution to the TCA cycle and energy metabolism, and its assistance in ammonia detoxification in the liver demonstrate the importance of GDH in liver biochemistry [13]. In this context, it has been predicted that serum levels of GDH may reflect liver tissue-related diseases, metabolic imbalances, and mitochondrial dysfunction [21]. In the studies conducted, serum nuclear DNA, mitochondrial DNA, and GDH levels were measured in patients with acute liver failure about mitochondrial dysfunction. ROC curve analysis results have shown that the combined evaluation of liver function tests, such as GDH (AUC: 0.70) and ALT (AUC: 0.59), may be clinically beneficial [24]. The capacity of GDH to reflect liver damage has also been evaluated in comparison with classical liver enzymes such as ALT and AST. [14, 24, 25]. The fact that ALT and AST are also expressed in muscle tissue limits their diagnostic specificity, particularly in cases accompanied by myopathies or drug-induced muscle damage. In this context, the study demonstrated that GDH can detect hepatocellular damage with high sensitivity and specificity, independent of muscle tissue-derived interference; therefore, it has been proposed as an alternative liver-specific biomarker to ALT. [25]. Another study in patients with Duchenne muscular dystrophy showed that ALT levels were deceptively elevated due to muscle damage, whereas GDH levels remained within normal limits and increased significantly when liver damage developed [14]. A study conducted on patients with drug-induced liver injury reported that GDH levels were not only diagnostic but also a prognostic marker associated with mortality. It was emphasized that GDH demonstrated high diagnostic accuracy in distinguishing drug-induced liver injury with an AUC value of 0.936 [26]. In our study, we also observed that, in addition to classical liver enzymes such as ALT, AST, and ALP, GDH levels were significantly elevated in cases with hepatitis B infection. ROC analysis revealed an AUC value of 0.658 for GDH, while the corresponding values for ALT and AST were 0.662 and 0.536, respectively. These results indicate that GDH provides a significant diagnostic value in reflecting hepatocellular damage and performs better compared to AST.

In recent studies, it has been reported that GDH, which plays a key role in glutamate metabolism, is involved in the regulation of tumor proliferation. [12, 27, 28]. High preoperative serum GDH levels have been shown to be significantly associated with microvascular invasion and poor prognosis after liver transplantation in HCC patients. It has been reported that GDH can provide a predictive value for the presence of microvascular invasion [12]. In another study demonstrating the prognostic role of GDH in tumor biology, it was reported that high GDH expression detected immunohistochemically was associated with poor prognosis and could be a potential therapeutic target in extrahepatic cholangiocarcinoma by affecting Smad-mediated signaling pathways [27]. A study on gastric cancer cells showed that GDH was expressed in the cytoplasm, membrane, and nucleus regions of tumor cells, and silencing the GDH gene significantly reduced cell proliferation, invasion, and migration [28]. A large-scale study on breast cancer subtypes reported that GDH expression was particularly high in luminal/ER + tumors, and that this high expression was significantly associated with better patient survival. Furthermore, it was emphasized that high GDH levels were associated with a better response to chemotherapy in triple-negative breast cancer patients [23]. In our study, newly diagnosed HCC patients exhibited significantly elevated GDH levels compared to the control group, in association with fibrosis and tumor formation. GDH demonstrated high diagnostic performance with an AUC of 0.808. These findings indicate that GDH may serve as a valuable biomarker for HCC diagnosis, reflecting both hepatic injury and tumor progression.

In the diagnosis and staging of liver diseases, the use of not only classical liver enzyme levels such as ALT, AST and ALP, but also biochemical ratios and indices such as APRI and AAR derived from these parameters increases the reliability of clinical evaluations [10, 29, 30]. Evaluating the degree of liver fibrosis in combination with these noninvasive biochemical indices facilitates monitoring of disease progression and prevents unnecessary invasive procedures [30, 31]. In a study conducted on NAFLD patients, it was reported that APRI showed high performance in terms of both sensitivity (84.1%) and specificity (88.2%) in detecting advanced fibrosis, while FIB-4 attracted attention with its particularly high sensitivity rate (97.7%) [29]. In another study conducted in HBV patients, it was reported that FIB-4 (AUC: 0.716) and APRI (AUC: 0.711) scores provided significant diagnostic accuracy in the diagnosis of advanced fibrosis, whereas the AAR score did not give statistically considerable discrimination [30]. In a study conducted in individuals with HBV-associated fatty liver disease, liver stiffness measured by FibroScan had the highest diagnostic accuracy (AUC: 0.851) in the diagnosis of fibrotic nonalcoholic steatohepatitis, whereas other noninvasive tests such as NFS (AUC: 0.725), FibroScan-AST score (AUC: 0.650) and FIB-4 (AUC: 0.560) showed lower accuracy [31]. In our study, patients with chronic HBV infection exhibited marked increases in GDH, AAR, FIB-4, and APRI levels as the fibrosis stage progressed. In HCC cases, significant elevations in these parameters were also observed at the advanced fibrosis stage (F4). Moreover, in the HCC group, noninvasive scores such as AAR (AUC: 0.929), APRI (AUC: 0.976), and FIB-4 (AUC: 1.000) demonstrated higher AUC values compared with those obtained in the HBV group (0.699, 0.701, and 0.811, respectively). These findings indicate that these noninvasive indices serve as reliable diagnostic tools reflecting disease severity in both HBV and HCC populations.

In our previous study, we demonstrated that oxidative stress markers, including increased MDA, TOS, and OSI levels and decreased GSH, SOD, and thiol parameters, were significantly altered in patients with HBV and HCC, reflecting a disrupted redox balance [20]. The present findings complement these results by showing that non-invasive fibrosis indices and GDH levels increase in parallel with fibrosis progression and disease severity. Taken together, these studies suggest that oxidative stress and impaired antioxidant defense may contribute to hepatocellular injury and fibrogenesis, which are reflected by both biochemical indices and fibrosis scores. Therefore, the combined evaluation of redox status and non-invasive fibrosis markers may provide a more comprehensive approach for assessing disease progression in HBV and HCC patients.

## Conclusion

While GDH is known to be an important metabolic enzyme in gastrointestinal diseases, its utility as a potential biomarker in the diagnosis of HBV infection and hepatocellular carcinoma, as well as its relationship with liver fibrosis and noninvasive scoring systems, has not been sufficiently elucidated. In this study, GDH levels were evaluated for the first time together with liver fibrosis stages, APRI, AAR, and FIB-4 indices, and AFP levels within the context of the possible molecular links between viral hepatitis and HCC. The findings demonstrated that serum GDH levels were significantly elevated in HCC cases and exhibited strong diagnostic performance. Moreover, in both HBV and HCC patients, noninvasive indices such as APRI, FIB-4, and AAR increased in parallel with liver stiffness values obtained by FibroScan. In HBV patients, positive correlations were also identified between the FIB-4 and APRI indices and AST, GGT, and LDH levels. Taken together, these results suggest that GDH, when assessed alongside noninvasive fibrosis indices such as AAR, FIB-4, and APRI, may provide a sensitive and reliable approach for evaluating liver injury and fibrosis severity.

## Declarations

### Data availability

The datasets used and/or analyzed during the current study are available from the corresponding author upon reasonable request.
